# A tradeoff between the losses caused by computer viruses and the risk of the manpower shortage

**DOI:** 10.1371/journal.pone.0191101

**Published:** 2018-01-25

**Authors:** Jichao Bi, Lu-Xing Yang, Xiaofan Yang, Yingbo Wu, Yuan Yan Tang

**Affiliations:** 1 School of Software Engineering, Chongqing University, Chongqing, 400044, China; 2 Faculty of Electrical Engineering, Mathematics and Computer Science, Delft University of Technology, Delft, GA 2600, The Netherlands; 3 Department of Computer and Infomation Science, The University of Macau, Macau; Victoria University, AUSTRALIA

## Abstract

This article addresses the tradeoff between the losses caused by a new virus and the size of the team for developing an antivirus against the virus. First, an individual-level virus spreading model is proposed to capture the spreading process of the virus before the appearance of its natural enemy. On this basis, the tradeoff problem is modeled as a discrete optimization problem. Next, the influences of different factors, including the infection force, the infection function, the available manpower, the alarm threshold, the antivirus development effort and the network topology, on the optimal team size are examined through computer simulations. This work takes the first step toward the tradeoff problem, and the findings are instructive to the decision makers of network security companies.

## 1 Introduction

Computer networks and online social networks provide us with a fast channel of acquiring information and communicating ideas. Meanwhile, computer viruses can also spread rapidly through these networks, inflicting enormous economic losses [1]. For the loss estimation, see Refs. [2–6]. When a new computer virus emerges, there is often no ready-made antivirus that is capable of detecting and eliminating it. As a result, before an antivirus targeting the virus is released, the virus is able to spread itself freely through networks, infecting a significant fraction of the hosts.

Consider a network security company that is dedicated to developing antiviruses. Suppose that when the fraction of the victims of a new virus exceeds a presupposed alarm threshold, the company will initiate a project of developing an antivirus against the virus. First, the amount of effort needed for the project, which is typically measured by persons years or persons months, is estimated [7–10]. When the effort is determined, the company will organize a team for the project. At this point, the decision maker of the company must make a decision on the size of the team. Definitely, the losses inflicted by the virus should be minimized. For this purpose, the development cycle for the project should be minimized or, equivalently, the number of the team members should be maximized. However, if too many manpower resources are injected into the project, the company will take the risk of having no enough manpower to undertake other projects. Therefore, a deliberate tradeoff must be made between the two conflicting demands of reducing the losses caused by the virus and reducing the team size. In our opinion, the tradeoff problem is worthy of deep-going study. To our knowledge, to date this problem has not been addressed mathematically.

The key to solving the tradeoff problem is to accurately estimate the speed and extent of virus infections. Computer virus spreading dynamics as an emerging interdiscipline is devoted to gaining insight into the consequence of computer viruses through modeling and analyzing their spreading process. Since the seminal work by Kephart and White [11, 12], large numbers of computer virus spreading models, ranging from the population-level spreading models [13–17] and the network-level spreading models [18–22] to the individual-level spreading models [23–30], have been proposed. In particular, a special type of spreading models known as the Susceptible-Infected (SI) models [31, 32] are especially suited to capturing the spreading process of a new digital virus before the relevant antivirus is released.

This article addresses the above-mentioned tradeoff problem. First, an individual-level virus spreading model, which is known as the individual-level SI model, is proposed to capture the spreading process of the virus before the appearance of its natural enemy, which is then utilized to assess the expected losses caused by the virus during the development period of an antivirus aiming at the virus. Then, the tradeoff problem is modeled as a discrete optimization problem. On this basis, the influences of different factors, including the infection force, the infection function, the available manpower, the alarm threshold, the antivirus development effort and the network topology, on the optimal team size are examined through computer simulations. This work takes the first step toward the tradeoff problem, and the findings are instructive to the decision makers of network security companies.

The subsequent materials of this work are organized as follows. Section 2 presents the individual-level SI model, and models the tradeoff problem. Section 3 experimentally examines the influences of different factors on the optimal team size. Finally, this work is summarized by Section 4.

## 2 The modeling of the tradeoff problem

Imagine that a network security company prepares to develop the antivirus aiming at a new computer virus. From the company’s perspective, the losses inflicted by the virus should be minimized, and the manpower allocated for the development project should be minimized so that there is enough manpower to undertake other projects. Therefore, the decision maker of the company must make a tradeoff between the two conflicting demands. This section is dedicated to modeling the tradeoff problem. For this purpose, the virus spreading process must first be modeled.

### 2.1 The modeling of the virus spreading process

Suppose the new virus appears at time *t* = 0 and then spreads through a network *G* = (*V*, *E*) connecting *N* hosts labelled 1, 2, ⋯*N*. Let **A** = (*a*_*ij*_)_*N*×*N*_ denote the adjacency matrix of the network. Before the release of the relevant antivirus, the virus is able to spread freely through the network, and every host in the network is either *susceptible* or *infected*. Let *X*_*i*_(*t*) = 0 and 1 denote the event that at time *t*, host *i* is susceptible and infected, respectively. Let *S*_*i*_(*t*) and *I*_*i*_(*t*) denote the probability of host *i* being susceptible and infected at time *t*, respectively.

$$\begin{array}{c} S_{i}\left(t\right)=\mathrm{Pr}\left\{X_{i}\left(t\right)=0\right\},\;I_{i}\left(t\right)=\mathrm{Pr}\left\{X_{i}\left(t\right)=1\right\}. \end{array}$$

Let *θ* denote the presupposed alarm threshold for the virus, *τ* the time at which the expected fraction of the infected hosts in the network exceeds *θ*.

$$\begin{array}{c} \tau =\inf\{t:\frac{1}{N}\sum_{i=1}^{N}I_{i}\left(t\right)\geq \theta \}. \end{array}$$ (1)

At this time, the security company will initiate the development project of the antivirus against the virus. Let *W* denote the effort of the project, *n* the number of the team members assigned to the project. Then the development period for the project is $\frac{W}{n}$.

It is assumed that due to the infections by neighboring infected hosts, at time $t\in [0,\tau +\frac{W}{n})$ susceptible host *i* gets infected at rate $\beta f(\sum_{j=1}^{N}a_{ij}I_{j}\left(t\right))$, where the parameter *β* > 0 is referred to as the *infection force*, the function *f* is referred to as the *infection function*, which is strictly increasing and concave, *f*(0) = 0, *f*(*x*) ≤ *x*, *x* ≥ 0. For the rationality of the assumption, see Ref. [30]. According to the assumption, the spreading process of the virus is modeled as the following dynamical system.

$$\begin{array}{c} \frac{dI_{i}\left(t\right)}{dt}=\beta \left[1-I_{i}\left(t\right)\right]f(\sum_{j=1}^{N}a_{ij}I_{j}\left(t\right)),\;0\leq t<\tau +\frac{W}{n},1\leq i\leq N. \end{array}$$ (2)

We refer to the model as the *individual-level SI model*.

### 2.2 The modeling of the tradeoff problem

Suppose the losses per unit time led by an infected host are one unit. Then the overall losses caused by the virus in the time interval $\left[\tau ,\tau +\frac{W}{n}\right)$ are expected to be

$$\begin{array}{c} L\left(n\right)=\sum_{i=1}^{N}\int_{\tau }^{\tau +\frac{W}{n}}I_{i}\left(t\right)dt. \end{array}$$ (3)

Definitely, this expected loss should be minimized, which implies that *n* should be maximized. However, with the increase of *n*, the company will take a higher risk of having no enough manpower to undertake other projects. To reduce the risk, *n* should be minimized. To the extreme, it is best to assign only a single person for the project. Therefore, the decision maker of the company must make a deliberate tradeoff between the two conflicting demands. Let $\bar{n}$ be the number of currently available programmers of the company. Let us measure the tradeoff with

$$\begin{array}{c} J\left(n\right)=kn+L\left(n\right)=kn+\sum_{i=1}^{N}\int_{\tau }^{\tau +\frac{W}{n}}I_{i}\left(t\right)dt, \end{array}$$ (4)

where *k* > 0 stands for the relative weight of the two parts in the tradeoff; a larger *k* value means an emphasis on the reduction of the risk of manpower shortage, whereas a smaller *k* value implies that a lower loss is pursued. The tradeoff problem is then reduced to solving the following discrete optimization problem.

$$\begin{array}{c} \text{Minimize}{\;}_{1\leq n\leq \bar{n}}J\left(n\right)=kn+\sum_{i=1}^{N}\int_{\tau }^{\tau +\frac{W}{n}}I_{i}\left(t\right)dt. \end{array}$$ (5)

An optimal solution to the optimization problem stands for a better choice of the team size from the company’s respective.

## 3 The determination of the factors involved in the optimization problem

The optimal team size, i.e., the optimal solution to the optimization problem (5), involves six factors: the network *G*, the infection force *β*, the infection function *f*, the alarm threshold *θ*, the antivirus development effort *W*, and the available manpower $\bar{n}$. Before solving the problem, these factors must be determined.

The available manpower $\bar{n}$ is at hand, the alarm threshold *θ* can be set flexibly by the company, the development effort can be estimated with the software cost estimation techniques given in Refs. [7–10], and the topological structure of the network *G* is obtainable using the network crawler described in Ref. [33].

The infection function *f* can be approached by applying the deep learning techniques presented in Refs. [34, 35] to the massive synthetic infection data. This is what we are going after.

The infection force *β* can be estimated by applying the time series analysis techniques exhibited in Ref. [36] to the successively monitored fraction of the infected hosts. See Refs. [37, 38]. This is what we will figure out.

When these factors are all determined, the optimization problem can be solved numerically.

**Fig 1:**
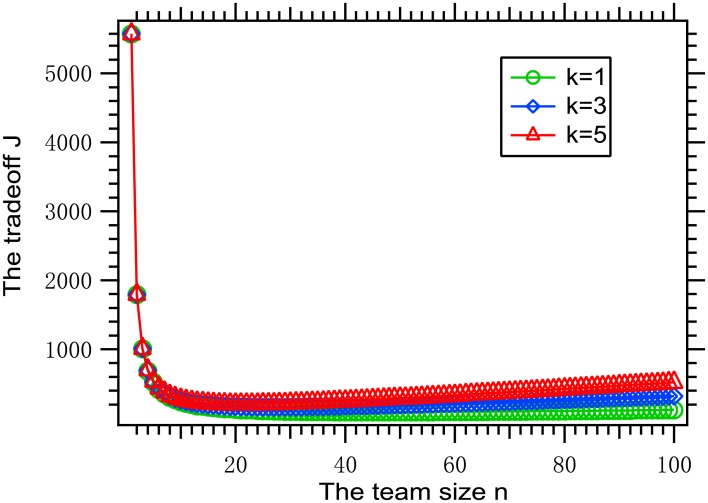
The team size vs. the tradeoff for the above parameters.

Consider three instances of the optimization problem (5), where *k* ∈ {1, 3, 5}, *β* = 0.001, $f\left(x\right)=\frac{x}{1+x}$, *θ* = 0.01, *W* = 100, $\bar{n}=100$, and a Facebook sub-network with 2000 nodes given in Ref. [39] is taken as the virus-spreading network, denoted *G*_0_. The team size vs. the tradeoff is shown in Fig 1. It can be seen that with the increase of the team size, the tradeoff first goes sharply down then goes slowly up, and the respective optimal team sizes are 47, 27 and 21 for *k* = 1, 3 and 5.

## 4 The influence of different factors on the optimal team size

The optimal team size is dependent upon six factors: the infection force *β*, the infection function *f*, the available manpower $\bar{n}$, the alarm threshold *θ*, the development effort *W*, and the network *G*. This section is devoted to exploring the influence of each of these factors on the optimal team size.

In the following five experiments, *G* = *G*_0_, the infection function $f\in \{f_{i}:f_{i}\left(x\right)=\frac{x}{1+ix},1\leq i\leq 5\}$.

### 4.1 The influence of the infection force

**Fig 2:**
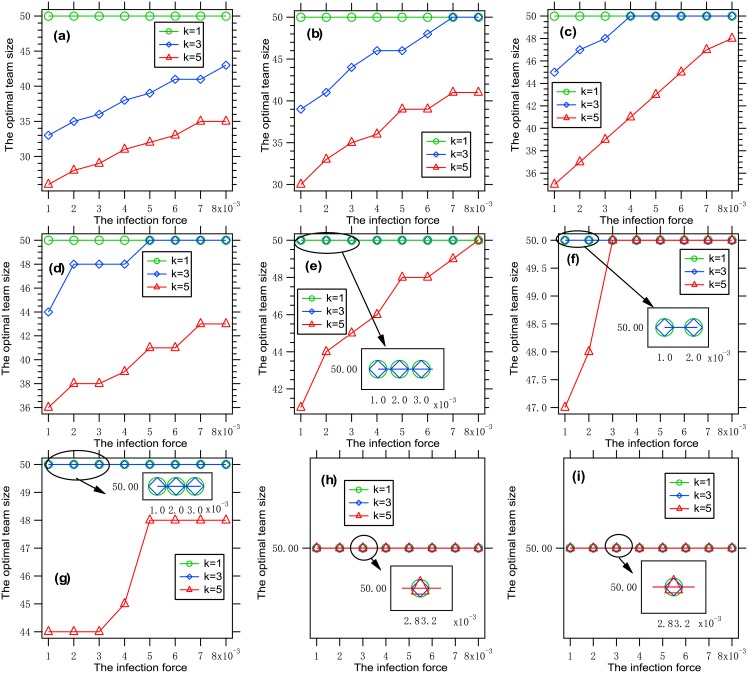
The optimal team size vs. the infection force. Each data point is obtained by solving the optimization problem (5) with *β* ∈ {*a* ⋅ 10^−3^: *a* = 1, ⋯, 8}, *k* ∈ {1, 3, 5}, *G* = *G*_0_, *f* = *f*_1_, $\bar{n}=50$, (a) *θ* = 0.01, *W* = 150; (b) *θ* = 0.01, *W* = 200; (c) *θ* = 0.01, *W* = 250; (d) *θ* = 0.02, *W* = 150, (e) *θ* = 0.02, *W* = 200; (f) *θ* = 0.02, *W* = 250; (g) *θ* = 0.03, *W* = 150; (h) *θ* = 0.03, *W* = 200; (i) *θ* = 0.03, *W* = 250. It can be seen that the optimal team size is increasing with the infection force.

To understand the influence of the infection force on the optimal team size, we present Fig 2, where each data point is obtained by solving the optimization problem (5) with a given set of parameters.

It is concluded from the figure that the optimal team size is increasing with the infection force. This phenomenon can be explained as follows. The loss part in the tradeoff is increasing with the infection force. To better balance the two parts in the tradeoff, the team size must be increased properly.

### 4.2 The influence of the infection function

**Fig 3:**
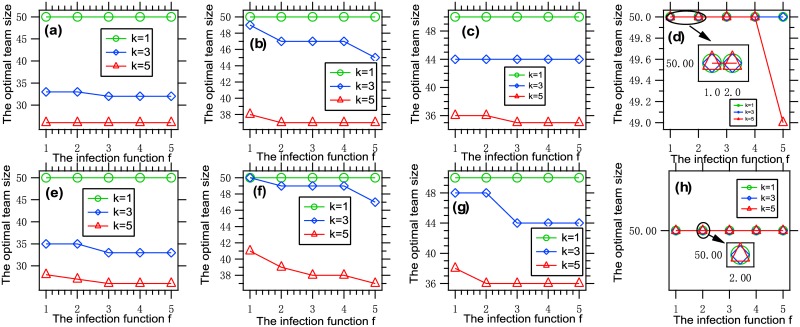
The optimal team size vs. the infection function. Each data point is obtained by solving an optimization problem with *f* ∈ {*f*_*i*_: 1 ≤ *i* ≤ 5}, *k* ∈ {1, 3, 5}, *G* = *G*_0_, $\bar{n}=50$, (a) *β* = 0.001, *θ* = 0.01, *W* = 150; (b) *β* = 0.001, *θ* = 0.01, *W* = 300; (c) *β* = 0.001, *θ* = 0.02, *W* = 150; (d) *β* = 0.001, *θ* = 0.02, *W* = 300, (e) *β* = 0.002, *θ* = 0.01, *W* = 150; (f) *β* = 0.002, *θ* = 0.01, *W* = 300; (g) *β* = 0.002, *θ* = 0.02, *W* = 150; (h) *β* = 0.002, *θ* = 0.02, *W* = 300. It can be seen that the optimal team size is increasing with the infection function.

To understand the influence of the infection function *f* on the optimal team size, we present Fig 3, where each data point is obtained by solving an optimization problem (5) with a given set of parameters.

It is concluded from the figure that the optimal team size is increasing with the infection function. The explanation of this phenomenon is similar to that of the previous one.

### 4.3 The influence of the available manpower

**Fig 4:**
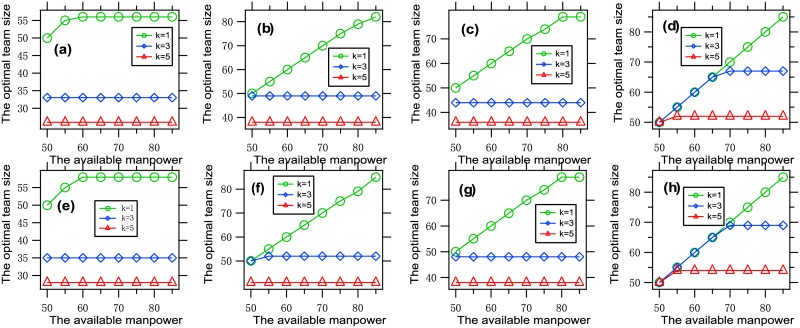
The available manpower vs. the optimal team size. Each data point is obtained by solving the optimization problem (5) $\bar{n}\in \left\{50,55,60,65,70,75,80,85\right\}$, *k* ∈ {1, 3, 5}, with *f* = *f*_1_, *G* = *G*_0_, (a) *β* = 0.001, *θ* = 0.01, *W* = 150; (b) *β* = 0.001, *θ* = 0.01, *W* = 300; (c) *β* = 0.001, *θ* = 0.02, *W* = 150; (d) *β* = 0.001, *θ* = 0.02, *W* = 300, (e) *β* = 0.002, *θ* = 0.01, *W* = 150; (f) *β* = 0.002, *θ* = 0.01, *W* = 300; (g) *β* = 0.002, *θ* = 0.02, *W* = 150; (h) *β* = 0.002, *θ* = 0.02, *W* = 300. It can be seen that the optimal team size is increasing and tends to saturation with the available manpower.

To understand the influence of the available manpower on the optimal team size, we present Fig 4, where each data point is obtained by solving the optimization problem (5) with a set of given parameters.

It is concluded from the figure that the optimal team size is increasing and tends to saturation with the available manpower. This phenomenon can be explained as follows. When there is a small available manpower, the balance between the two parts in the tradeoff would lead to an optimal team size that is equal to the available manpower. With the increase of the available manpower, the balance would lead to an optimal team size that is increasing less rapidly than the available manpower and finally tends to saturation.

### 4.4 The influence of the alarm threshold

**Fig 5:**
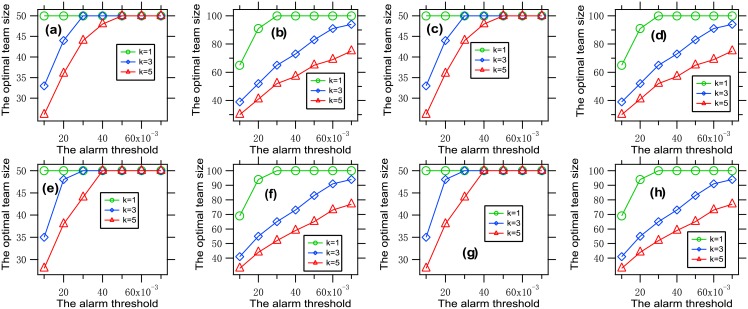
The optimal team size vs. the alarm threshold. Each data point is obtained by solving the optimization problem (5) with *θ* ∈ {*a* ⋅ 10^−2^: *a* = 1, ⋯, 7}, *k* ∈ {1, 3, 5}, *f* = *f*_1_, *G* = *G*_0_, (a) *β* = 0.001, $\bar{n}=50$, *W* = 150; (b) *β* = 0.001, $\bar{n}=100$, *W* = 200; (c) *β* = 0.001, $\bar{n}=50$, *W* = 150; (d) *β* = 0.001, $\bar{n}=100$, *W* = 200, (e) *β* = 0.002, $\bar{n}=50$, *W* = 150; (f) *β* = 0.002, $\bar{n}=100$, *W* = 200; (g) *β* = 0.002, $\bar{n}=50$, *W* = 150; (h) *β* = 0.002, $\bar{n}=100$, *W* = 200. It can be seen that the optimal team size is increasing with the alarm threshold.

To understand the influence of the alarm threshold on the optimal team size, we present Fig 5, where each data point is obtained by solving the optimization problem (5) with a given set of parameters.

It is concluded from this figure that the optimal team size is increasing with the alarm threshold. This phenomenon can be explained as follows. The loss part in the tradeoff is increasing with the alarm threshold. To better balance the two parts in the tradeoff, the team size must be increased properly.

### 4.5 The influence of the antivirus development effort

**Fig 6:**
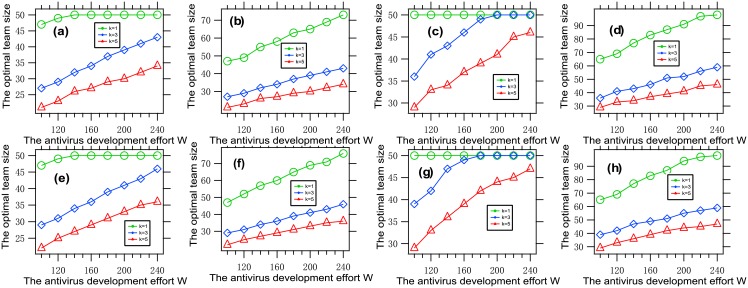
The optimal team size vs. the antivirus development effort. Each data point is obtained by solving the optimization problem (5) with *W* ∈ {80 + *a* ⋅ 20: *a* = 1, ⋯, 8}, *k* ∈ {1, 3, 5}, *f* = *f*_1_, *G* = *G*_0_, (a) *β* = 0.001, *θ* = 0.01, $\bar{n}=50$; (b) *β* = 0.001, *θ* = 0.01, $\bar{n}=100$; (c) *β* = 0.001, *θ* = 0.02, $\bar{n}=50$; (d) *β* = 0.001, *θ* = 0.02, $\bar{n}=100$, (e) *β* = 0.002, *θ* = 0.01, $\bar{n}=50$; (f) *β* = 0.002, *θ* = 0.01, $\bar{n}=100$; (g) *β* = 0.002, *θ* = 0.02, $\bar{n}=50$; (h) *β* = 0.002, *θ* = 0.02, $\bar{n}=100$. It can be seen that the optimal team size is increasing with the antivirus development effort.

To understand the influence of the antivirus development effort on the optimal team size, we present Fig 6, where each data point is obtained by solving an optimization problem with a given set of parameters.

It is concluded from this figure that the optimal team size is increasing with the antivirus development effort. This phenomenon can be explained as follows. The loss part in the tradeoff is increasing with the effort. To better balance the two parts in the tradeoff, the team size must be increased properly.

### 4.6 The influence of the network heterogeneity

**Fig 7:**
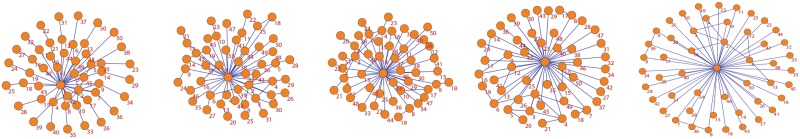
Five scale-free networks with 100 nodes, 109 edges, and a power exponent of 2.7, 2.8, 2.9, 3.0, and 3.1, respectively.

To understand the influence of the network heterogeneity on the optimal team size, the following experiment assumes *G* ∈ {*G*_*i*_: 1 ≤ *i* ≤ 5}, where *G*_*i*_ are scale-free networks with 100 nodes, 109 edges, and a power exponent of 2.7, 2.8, 2.9, 3.0, and 3.1, respectively [40]. See Fig 7.

**Fig 8:**
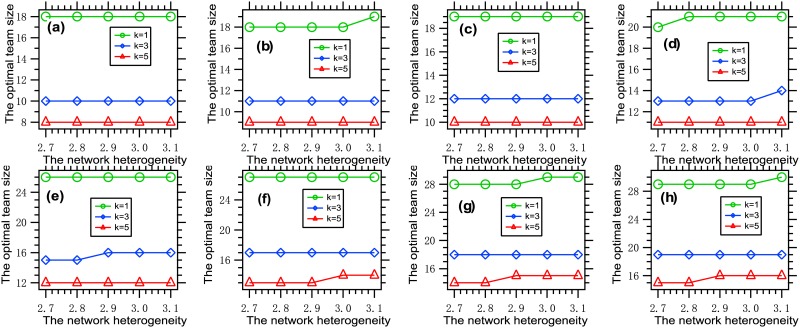
The optimal team size vs. the network heterogeneity. Each data point is obtained by solving the optimization problem (5) with *G* ∈ {*G*_*i*_: 1 ≤ *i* ≤ 5}, *k* ∈ {1, 3, 5}, *f* = *f*_1_, *W* = 300, $\bar{n}=50$ (a) *β* = 0.001, *θ* = 0.01; (b) *β* = 0.003, *θ* = 0.01; (c) *β* = 0.005, *θ* = 0.01; (d) *β* = 0.007, *θ* = 0.01; (e) *β* = 0.002, *θ* = 0.02; (f) *β* = 0.004, *θ* = 0.02; (g) *β* = 0.006, *θ* = 0.02; (h) *β* = 0.008, *θ* = 0.02. It can be seen that the optimal team size is increasing with the network heterogeneity.

We present Fig 8, where each data point is obtained by solving the optimization problem (5) with a given set of parameters.

It is concluded from this figure that the optimal team size is increasing with the network heterogeneity. This phenomenon can be explained as follows. The loss part in the tradeoff is increasing with the effort, because malware spreads more rapidly in a more heterogeneous network than in a more homogeneous network. To better balance the two parts in the tradeoff, the team size must be increased properly.

## 5 Conclusions

This article has addressed the tradeoff between the losses caused by a new virus and the size of the team for developing an antivirus against the virus. First, an individual-level virus spreading model has been proposed to capture the spreading process of the virus before the appearance of its natural enemy. Then, the tradeoff problem is modeled as an optimization problem. Next, the influences of different factors, including the infection force, the infection function, the available manpower, the alarm threshold, the antivirus development effort and the network topology, on the optimal team size have been examined through computer simulations. The findings are instructive to the decision makers of network security companies.

Towards this direction, there are a number of problems that are worth study. As was indicated previously, the infection force and the infection function must be determined. The model should be extended to more sophisticated virus spreading models such as the impulsive spreading models [41–43], the stochastic spreading models [44–46], and the spreading models on time-varying networks [47–49].
